# RCVS: by clinicians for clinicians—a narrative review

**DOI:** 10.1007/s00415-022-11425-z

**Published:** 2022-10-28

**Authors:** Deborah Katharina Erhart, Albert Christian Ludolph, Katharina Althaus

**Affiliations:** Department of Neurology, University of Ulm, Oberer Eselsberg 45, 89081 Ulm, Germany

**Keywords:** Reversible cerebral vasoconstriction syndrome, Posterior reversible encephalopathy syndrome, Thunderclap headache, Primary angiitis of the central nervous system

## Abstract

**Background/Objective:**

Reversible cerebral vasoconstriction syndrome may be underdiagnosed. It can be accompanied by various complications, mainly intracerebral hemorrhage and ischemic stroke. The clinical presentation of this condition varies according to its localization. The aims of this review are to raise awareness of the disease, especially in the presence of corresponding risk factors; to connect its precipitating factors, pathophysiology, and complications; and to compare various differential diagnoses of vasoconstriction.

**Methods:**

A review of the literature in PubMed/MEDLINE and Google Scholar was conducted from May 1997 until May 2022.

**Results:**

Reversible cerebral vasoconstriction syndrome, which is a clinical–radiological syndrome, is mainly characterized by the occurrence of thunderclap headache and widespread vasoconstriction. The most common precipitating factors are the use of vasoactive substances and postpartum status. The pathophysiology is currently assumed to include two mechanisms: sympathetic overactivity and endothelial dysfunction. From these mechanisms, it is possible to derive potential complications as well as the most important differential diagnoses: posterior reversible encephalopathy syndrome, convexity subarachnoid hemorrhage, ischemic and hemorrhagic stroke, and primary angiitis of the central nervous system.

**Conclusion:**

In general, the outcome of reversible cerebral vasoconstriction syndrome is very good. Vasospasm as well as thunderclap headache attacks can be fully reversible, and > 90% of patients are functionally independent at discharge.

**Supplementary Information:**

The online version contains supplementary material available at 10.1007/s00415-022-11425-z.

## Introduction

Reversible cerebral vasoconstriction syndrome (RCVS), whose cardinal symptom is acute, worst-ever headache (thunderclap headache (TCH)) in approximately 85% of cases, is mainly a clinical–radiological syndrome and may or may not include neurological deficits [1–3]. The clinical presentation often depends on complications such as ischemic stroke, intracranial hemorrhage, and posterior reversible encephalopathy syndrome. Depending on the affected area, aphasia, hemiparesis, hemianopsia, epileptic seizures, or Balint syndrome may occur in up to 43% of cases [4]. In > 85% of cases, a characteristic episode of TCH can be seen approximately 2–3 weeks prior [5]. Cranial imaging by computed tomography (CT) or magnetic resonance imaging (MRI) should be followed by vascular imaging with CT angiography or MR angiography [6]. Aside from the treatment of acute complications, such as stroke, intracranial hemorrhage, and epileptic seizures, the first-line drug for RCVS treatment is the calcium channel antagonist nimodipine [7–9]. It is administered first orally and then intra-arterially in fulminant cases [9, 10]. In 2007, Calabrese et al. characterized RCVS, for which there already existed multiple case reports and different terms (Table 1) [6, 11–28]. A detailed overview of RCVS cases characterized by risk factors/triggers and complications can be found in the “Supplementary Information” section (see Online Reference 1). Diagnostic criteria have also been established (Table 2) [11]. RCVS is mainly marked by widespread vasoconstriction from distal to proximal vascular segments, which regresses within 3 months and is mainly precipitated by various factors, such as vasoactive drug intake or postpartum status [11]. The pathophysiology of this syndrome is still not fully understood [29], but it is thought to encompass two main mechanisms: endothelial dysfunction and disturbances in cerebral vascular tone caused primarily by sympathetic overactivation [6, 12, 30–36].

**Table 1:** Different former terms for RCVS [6, 11–17]

Postpartum angiopathy (PPA)
Benign angiopathy of the central nervous system
Call-(Fleming) syndrome
Migraine angiitis
Migrainous vasospasm
Idiopathic thunderclap headache with reversible vasospasm
Drug-induced cerebral vasculopathy
Drug-induced cerebral angiitis
Fatal vasospasm in migrainous infarction
Sexual headache

**Table 2:** The main diagnostic criteria of RCVS, established by Calabrese et al., 2007 [11]

Conventional angiography (DSA)/CTA/(TOF-) MRA with widespread segmental cerebral artery vasoconstriction (“string-and-beads”)
No indications of an aneurysmal subarachnoid hemorrhage
Normal or almost normal cerebrospinal fluid (CSF) analysis (mild pleocytosis or slightly elevated protein level)
Acute and severe headache, with or without focal neurological deficits
Reversibility of angiographic abnormalities within 3 months (In case of death before completion of follow-up, autopsy should exclude appropriate differential diagnoses.)

The aim of this narrative review is to link the precipitating factors, pathophysiology and complications of RCVS. Another important point is the detailed analysis of possible differential diagnoses to prevent misdiagnosis and protect patients from unnecessary, risky diagnostics, and even harmful therapy.

## Methods

**Fig. 1:**
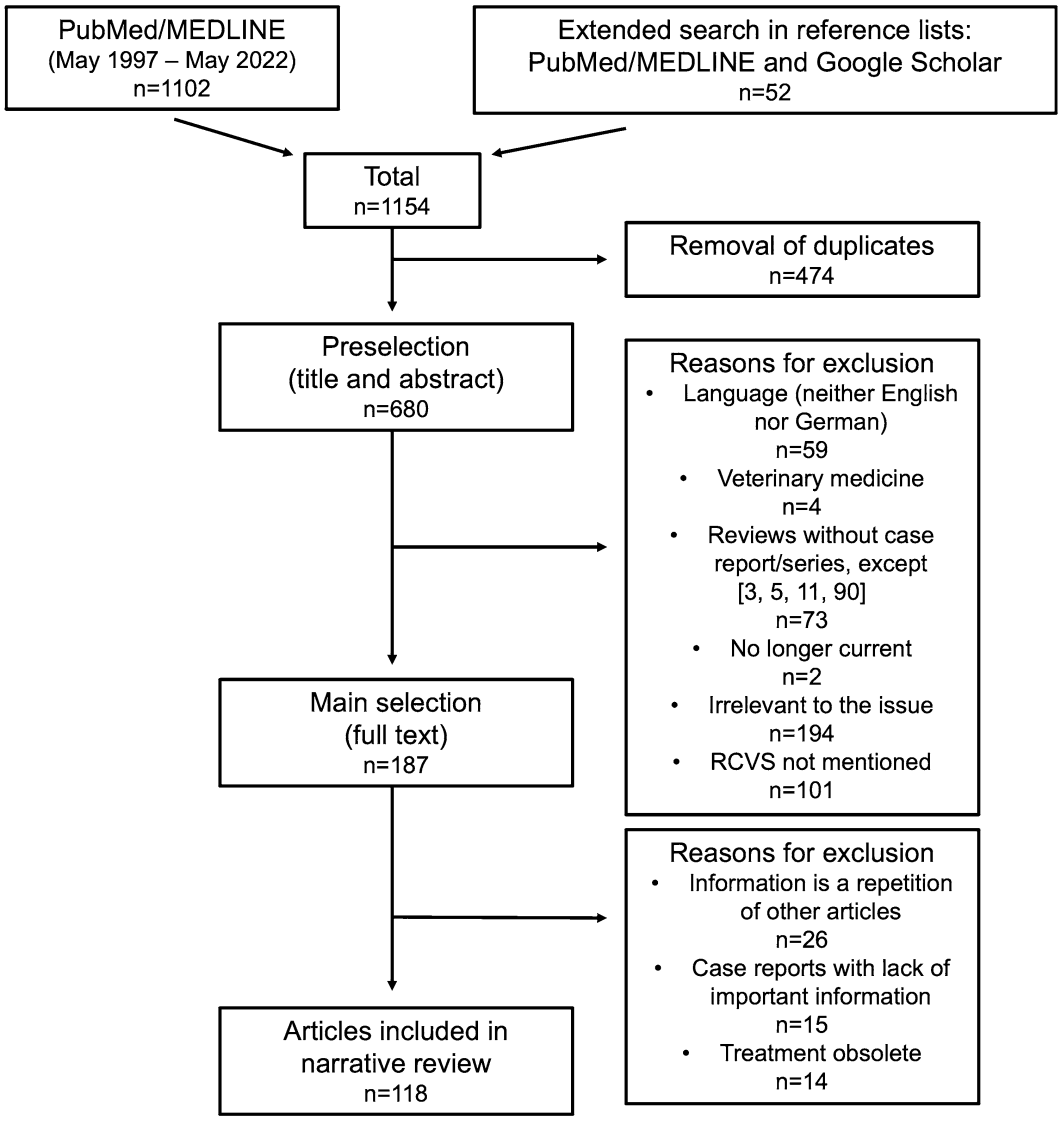
The literature search is illustrated in this flowchart. The literature search described under "Methods" identified 1154 articles that could potentially be considered. In a two-stage selection process using the exclusion criteria mentioned above, 118 articles were ultimately included in the review

The systematic literature search for this review encompassed the PubMed/MEDLINE and Google Scholar databases, and was performed by the first author (DKE). The search terms (in PubMed/MEDLINE) were "RCVS pathophysiology", “RCVS AND serotonergic drugs”, “RCVS and corticosteroids”, “RCVS AND thunderclap headache”, “RCVS AND postpartum”, "RCVS AND COVID-19", "RCVS AND stroke", “RCVS AND intracranial hemorrhage”, "RCVS AND PRES" (posterior reversible encephalopathy syndrome), "RCVS AND PACNS" (primary angiitis of the central nervous system), “RCVS AND treatment”, and “RCVS AND imaging”; articles published from May 1997 to May 2022 were considered. In addition, the search was extended to the bibliographies of the articles found with the terms mentioned above. PubMed/MEDLINE and Google Scholar were used for this purpose. Only articles published in English or German were included. The results of the literature search with the above-mentioned terms is shown in Table 3, and a flowchart of the literature search is shown in Fig. 1.

**Table 3:** Records found in systematic literature searches using the search criteria below in combination with "RCVS" in the PubMed/MEDLINE database from May 1997 to May 2022

Criteria	Number of articles found
Pathophysiology	118
PRES	64
Corticosteroids	8
Serotonergic drugs	7
Thunderclap headache	18
Postpartum	64
COVID-19	15
Stroke	144
Intracranial hemorrhage	109
PACNS	34
Imaging	240
Treatment	266
Digital subtraction angiography	16

*PRES* posterior reversible encephalopathy syndrome, *PACNS* primary angiitis of the central nervous system

## Results

### Epidemiology

The incidence of RCVS is unknown but steadily increasing. In general, RCVS can affect any age group, even adolescents and children, but the mean age is between 42 and 47 years [4, 37–39]. Depending on the study, the gender ratio varies from 1.8:1 to 8:1, with a female predominance [4, 6]. Explanations for the variability of the ratio may include the involvement of different diagnostic criteria and/or differences in recruiting centers (emergency room vs. headache clinic; outpatient vs. inpatient) [40, 41]. The ratio for secondary RCVS (caused by a risk factor) is slightly reduced [6]. On average, male patients are a decade younger than female patients (third-to-fourth decade vs. fourth-to-fifth decade, respectively) [42]. Surprisingly, in children/adolescents, up to 85% of patients are male [43]. Reasons for the discrepancy with adults have not yet been identified. Generally, in > 90% of cases, a risk factor has been identified [43–45].

### Risk factors and triggers

RCVS can occur spontaneously/idiopathically, without a precipitating factor; in other patients, it occurs secondarily (approximately 40–60% of cases) [1, 46]. The precipitating factors and triggers of RCVS and TCH, respectively, are manifold (Table 4), which illustrates why the exact pathophysiology is not yet fully understood [11, 12, 17, 47–61]. Trigger factors for the most severe headache in a patient’s life can include coughing, bathing, physical exertion, Valsalva maneuvers, and sexual activity [55, 62–65]. Headaches caused by trigger factors may represent a primary headache disorder [66]. However, when present in combination with focal neurological deficits and appropriate angiographic findings, these factors may be considered etiologically to be triggers of RCVS [19, 20, 55, 62–64]. According to two of the four main studies, the most frequent precipitating factors, accounting for 31% of cases overall, are vasoactive medications (especially serotonergic drugs) and the postpartum period [6, 67]. Vasoactive substances mainly include serotonergic drugs, such as selective serotonin reuptake inhibitors (SSRIs); serotonin–noradrenaline reuptake inhibitors (SNRIs); 3,4-methylenedioxymethamphetamine (ecstasy); and triptans and sympathomimetics with serotonergic effects, such as over-the-counter drugs for upper respiratory tract infections (e.g., pseudoephedrine), amphetamines, cocaine, and ergot derivatives [13, 14, 49, 50, 68–70]. The use of these substances in combination with each other or with cannabis or opioids (which also have known SSRI effects) has already led to fatal cases of RCVS [46, 53, 71–73]. Vasoactive drugs act via specific receptors (antidepressants: 5-HT1B and 5-HT2A; triptans: 5-HT1B and 5-HT1D) located on smooth muscle cells in the area of peripheral and central blood vessels, and serotonergic–sympathomimetic synergism can cause potent cerebral/myocardial vasoconstriction [4, 13, 14, 47]. An important precipitating factor for men is cannabis use (up to 32% in the French case series), whose ingredient tetrahydrocannabinol can trigger vasoconstriction in different models [46, 71, 72]. In up to 40% of RCVS cases, there is a history of migraine [4, 6, 8]. One reason may be that triptans are precipitating factors for RCVS [50].

**Table 4:** Various precipitating factors/conditions and triggers causing RCVS [3, 11–13, 17, 21–28, 33, 48–65]

Drugs Selective serotonin (and noradrenaline) reuptake inhibitors, triptans (sumatriptan), ergotamine, pseudoephedrine, cocaine, amphetamine derivatives, ecstasy, lysergic acid diethylamide, tetrahydrocannabinol, cyclophosphamide, fingolimod, tacrolimus, erythropoetine, intravenous immune globuline, red blood cell transfusion, prednisolone, oral contraceptive pills
Conditions Early and late pregnancy, pre-eclampsia, (postpartum) eclampsia Tumors: pheochromocytoma, neuroendocrine tumor (e.g., bronchial carcinoid) Traumatic brain injury, neurosurgical procedures Porphyria Vascular: post-carotid endarterectomy, unruptured cerebral aneurysm, spinal subdural hematoma COVID-19
Idiopathic
Triggers Laughing, coughing, bathing, Valsalva maneuver, exertion, emotion, sexual acitivity

RCVS in the postpartum period, previously known as postpartum angiopathy (PPA), usually peaks 2 weeks after delivery [42, 48]. Many case reports and smaller case series have reported the coexistence of widespread cerebral vasoconstriction and pronounced parieto-occipital vasogenic edema, also referred to as posterior reversible encephalopathy syndrome (PRES), in the postpartum state [12, 15, 35, 47, 74]. The presence of eclampsia is a major risk factor for the development of PRES (24–47%) [15]. In addition, there is also a close association of PRES with RCVS as a complication in 7–38% of cases [4, 6, 72]. According to the American College of Obstetricians and Gynecologists (ACOG), eclampsia is defined as pre-eclampsia with the occurrence of new seizures or coma [75]. In 25% of cases, it occurs in the postpartum period [47]. With acute severe headache or TCH, seizures, hypertension, and focal neurological deficits, RCVS and eclampsia share several common symptoms, mostly neurological [12, 35, 76–79]. Moreover, in more than half of RCVS patients postpartum, characteristic manifestations of eclampsia, e.g., hypertension and PRES, are observed [15]. With regard to clinical (seizures, hemiparesis, and visual deficits) and radiological (vasoconstriction, restricted diffusion, PRES) features, RCVS/PPA and eclampsia have some overlap [12, 15, 74]. One article also speaks of distinct spectra of a disease process with the same fundamental pathophysiology, namely, endothelial dysfunction, as will be discussed in more detail in the next section [15].

In summary, the previous explanations help us to understand why RCVS has such a female predominance [1, 4]. According to Topcuoglu et al., nonpregnant women with RCVS were, on average, older than male patients (48 ± 11 years vs. 34 ± 13 years) and had more underlying depression, antidepressant use, and migraine [42]. In the following, the underlying pathophysiology will be explained in more detail, with emphasis on hormonal differences between the sexes and their role in vasoconstriction.

#### Excursion: RCVS and COVID-19

A limited number of case reports and one small case series of RCVS related to COVID-19, including mild respiratory infection, have been published [52, 80–82]. In 30% of COVID-19 patients with RCVS, none of the previously known precipitants could be detected [80]. Enhanced activation of the classical renin–angiotensin system (RAS) axis with sympathetic overactivation, which may lead to vasoconstriction of cerebral vessels, was postulated to occur via downregulation of the angiotensin-converting enzyme 2 (ACE2) receptor directly by SARS-CoV-2 [52, 81, 83]. This mechanism, as well as a coagulopathic/proinflammatory state in the context of critical illness, is also known to lead to ischemia in the context of COVID-19 [84]. Treatment with nimodipine was successful in most cases [81]. The severity of COVID-19 infection was not indicative of the extent of RCVS and its potential complications [80, 81].

### Clinical presentation

**Fig. 2:**
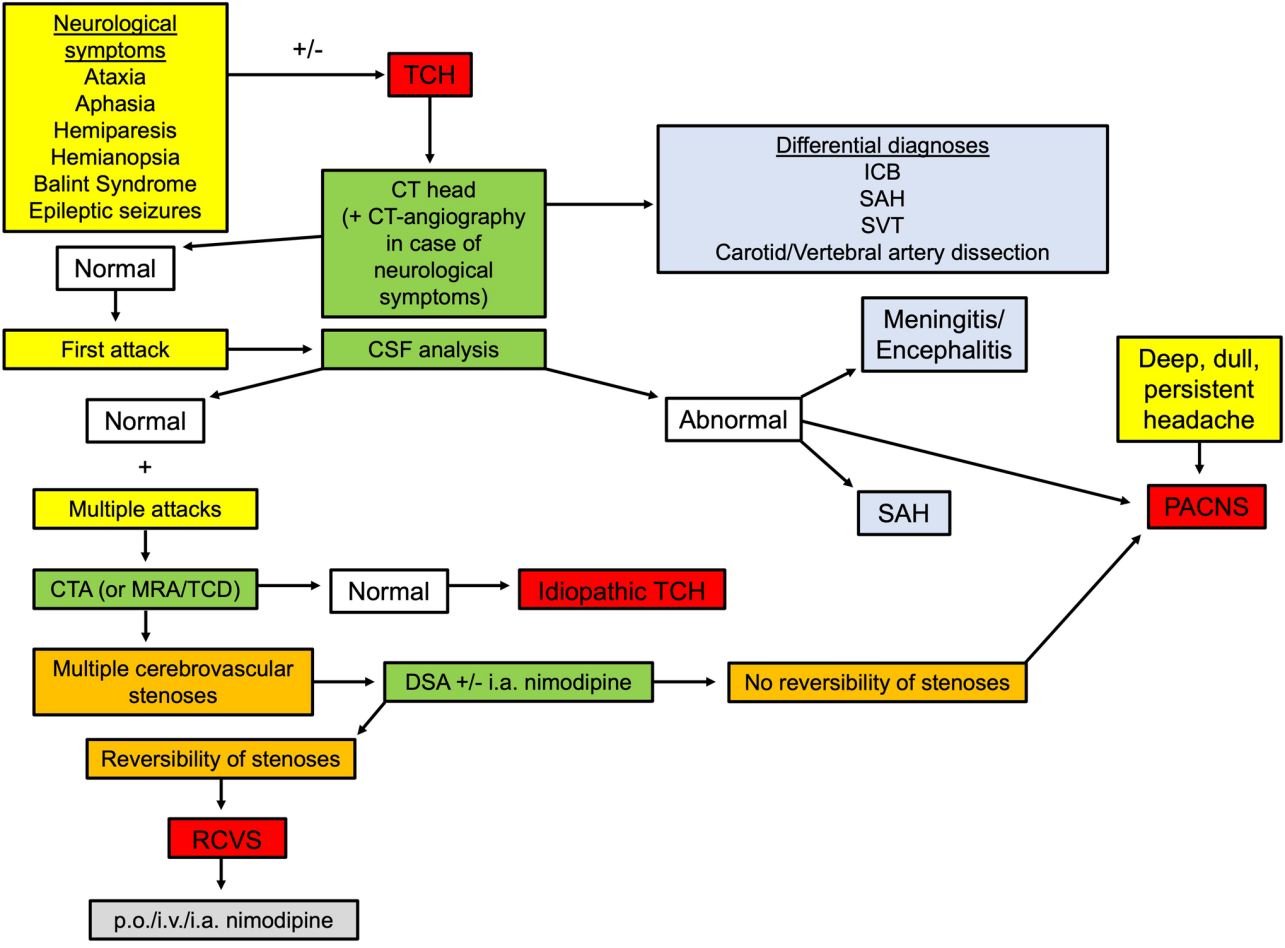
This flowchart shows the diagnostic workflow from thunderclap headache (TCH) as a leading symptom to the detection of reversible cerebral vasoconstriction syndrome (RCVS) [2, 3, 5, 7, 9, 11, 98, 99, 105, 110, 112, 114, 116]. *TCH* thunderclap headache, *CTA* computed tomography angiography, *MRA* magnetic resonance angiography, *TCD* transcranial doppler, *DSA* digital subtraction angiography, *ICB* intracerebral hemorrhage, *SAH* subarachnoid hemorrhage, *SVT* sinus venous thrombosis, *PACNS* primary angiitis of the CNS, *RCVS* reversible cerebral vasoconstriction syndrome, *CSF* cerebrospinal fluid, *i.v*. intravenous, *i.a*. intra-arterial, *p.o*. per os

First, it must be noted that in 85–100% of cases, RCVS is associated with recurrent episodes of TCH, that is, worst-ever headache that peaks in ≤ 1 min and is often accompanied by screaming and crying (32%), photophobia (30%), nausea (57%), and vomiting (38%) [5, 6, 38]. Characteristically, these episodes occur in clusters within up to 4 weeks and may or may not be accompanied by focal neurological deficits and/or epileptic seizures, as noted in the beta version of the International Classification of Headache Disorders 3 (ICHD-3) criteria [66]. In up to one-third of all patients, hypertensive blood pressure levels occur during the attacks [6]. In the time between TCH attacks, a slight, persistent headache may occur in approximately 35% of cases [6]. TCH is not pathognomonic for RCVS, because it may also be the first sign of other conditions: subarachnoid hemorrhage (SAH) due to ruptured aneurysm, cerebral sinus vein thrombosis, cervical artery dissection, pituitary apoplexy, ischemic/hemorrhagic stroke, colloid cyst of the third ventricle, or intracranial infection [5, 74]. Meanwhile, TCH itself is attributable to sudden vasodilatation activating the perivascular sensory nerve fibers of the dorsal root of C2 as well as the trigeminal nerve [6]. In up to 15% of cases, RCVS may occur without the characteristic TCH [18, 72, 85]. Strongly suggestive of the presence of RCVS are recurrent TCH episodes (80%) in combination with initial brain parenchymal imaging showing no lesions or intracranial hemorrhage (up to 55%) [4]. Primary and secondary TCH onset may be spontaneous or associated with the following triggers: bathing, coughing, Valsalva maneuvers, and physical exertion [5, 19, 20, 55, 62, 63]. Based on TCH as the leading symptom, Fig. 2 represents the diagnostic workflow with regard to clinical management primarily for RCVS, with attention to the differential diagnoses.

Focal neurological deficits (8–43% of cases) depend mainly on the complications of vasoconstriction [4, 40, 86]. Vasoconstriction even outlasts headache resolution and is therefore not a direct cause of headache [6, 40]. The focal deficits can also occur temporarily in the course of a transient ischemic attack (16%) [6]. Clinical symptoms depend primarily on the complications associated with RCVS, such as ischemic (39%) and hemorrhagic strokes (44%) and PRES (38%). The main presentations of PRES include encephalopathy (50–80%), seizures (60–75%), a dull, diffuse headache (50%), and visual disturbances (mainly Balint syndrome) (33%). These neurological symptoms are manifested acutely or subacutely, as in RCVS [76–79, 87]. However, the incidence of seizures in RCVS is lower, at 7–17% [4, 88]. Hemiparesis, hemianopsia, aphasia, dysarthria, and cortical blindness are often associated with intracranial hemorrhage and ischemic infarcts [4, 6, 23, 49, 57, 74].

### Pathophysiology

The pathophysiology of RCVS is not fully understood at present. Two main hypotheses are currently being discussed: alterations in cerebral vascular tone and endothelial dysfunction [31, 32, 34–36]. Cerebral blood flow usually remains constant and thus ensures cerebral circulation [89]. This is achieved by changes in the cerebral arterioles [90]. The process of cerebral blood flow autoregulation can be disturbed by certain factors, such as sympathomimetic drugs or dysfunction in the autonomic nervous system, which have been shown to result in increased sympathetic activity as well as a reduced parasympathetic response by analyses of heart rate variability in RCVS patients [32]. Cerebral blood vessels are richly innervated by sympathetic nerve fibers [32, 90]. Sympathetic overactivity in RCVS is evident from its major risk factors of vasoactive agents (such as sympathomimetic and serotonergic drugs) and hormone-secreting tumors such as pheochromocytoma, as well as the 33–47% prevalence of systolic blood pressure surges in patients presenting with RCVS [1, 6, 49, 68]. This hyperperfusion overwhelms the body’s capacity for vascular autoregulation [35]. It results in endothelial dysfunction and breakdown of the blood‒brain barrier, causing not only increased vascular permeability but also vasoconstriction in the middle and distal arterial branches (PRES) or delayed proximal vasoconstriction after 1–2 weeks in RCVS [12, 33]. The endothelium usually regulates cerebral vascular tone by secreting certain vasoconstrictors and vasodilators [90]. The close association of RCVS with PRES, which can occur as a complication in up to 38% of RCVS cases, suggests a common pathophysiology [4, 33, 91]. There are also case reports of both syndromes affecting the same patient with the same risk factors (e.g., pre-eclampsia) [12, 15, 74]. A factor that appears to play a critical role in the development of both PRES and RCVS is the breakdown of the blood‒brain barrier, which can be visualized by contrast-enhanced FLAIR (CE-FLAIR) MRI [34]. Sixty-nine percent of patients with definite RCVS and 25% of patients with probable RCVS showed breakdown of the blood‒brain barrier on CE-FLAIR-MRI, even without the appearance of PRES [34]. In addition, blood–brain barrier dysfunction may be intrinsic to the pathophysiology of RCVS development [34].

Oxidative stress, hormonal and biochemical factors, and genetic predisposition also play critical roles [31, 92]. Cerebral vessel walls contain gonadal hormone receptors [93, 94]. Estrogen modulates vascular tone and cerebral blood flow via both nongenomic and genomic pathways in cerebral arteries and arterioles. This is achieved through, among other things, reduction of sympathetic tone; stimulation of endothelial nitrite oxide synthase (eNOS) gene expression; eNOS phosphorylation; and a shift in the prostanoid balance in favor of prostacyclin (PGI2), which has vasodilatory effects [95]. Additionally, sex steroids modulate the permeability of the blood‒brain barrier (BBB), on which estrogens have a marked effect [42, 96]. This could explain why the postpartum period, with its accompanying drop in estrogen levels, is an important trigger for RCVS (and PRES) [42]. However, according to a study by Topcuoglu and Singhal, different female subgroups (e.g., postpartum, nonpregnant, premenopausal, and postmenopausal) did not differ in terms of clinical and radiological outcomes or severity of RCVS [42]. Nevertheless, childbirth remains an important risk factor for the development of RCVS [42, 74]. A crucial factor in the development of pre-eclampsia is the role of the placenta, which secretes inflammatory cytokines as well as the proangiogenic proteins placental growth factor, its soluble receptor, and soluble endoglin into the maternal circulation [35, 97]. Thus, a systemic immune response with endothelial dysfunction and vasoconstriction follows. This may result in both PRES and RCVS [35].

Moreover, genetic predisposition is a possibility for cases in which no trigger can be found. According to certain studies, a gene polymorphism (Val66Met) in the brain-derived neurotrophic factor (BDNF) gene increases the severity of vasoconstriction in RCVS [92].

### Complications and neuroimaging findings

**Fig. 3:**
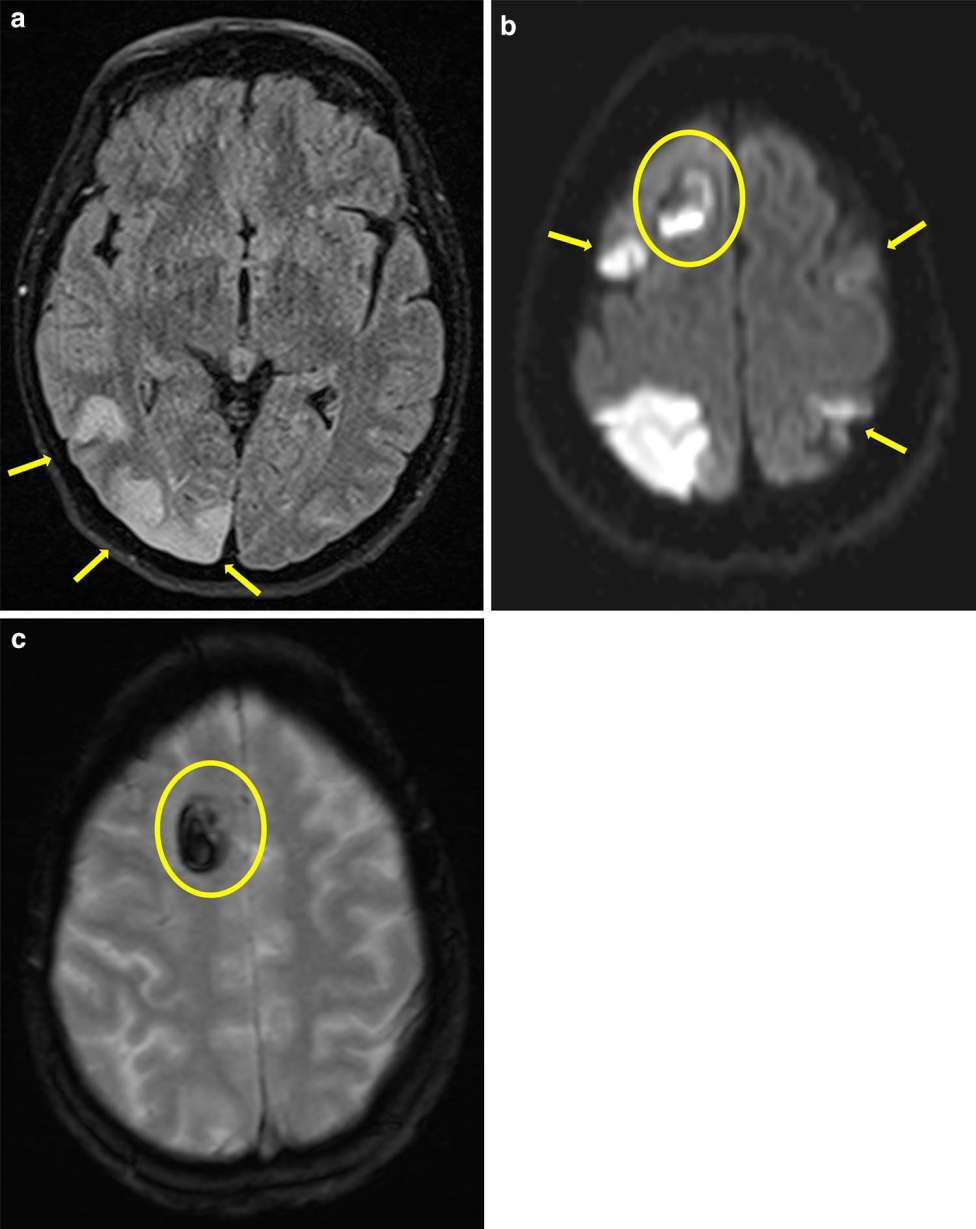
The following magnetic resonance imaging (MRI) findings were obtained in a 55-year-old female patient with reversible cerebral vasoconstriction syndrome (RCVS). They reflect the most common complications of RCVS. **a** Occipital, predominantly right-lateralized fluid-attenuated inversion recovery (FLAIR) hyperintensities (transverse; yellow arrows), consistent with posterior reversible encephalopathy syndrome (PRES). **b** Hyperintensities on diffusion-weighted imaging (DWI) and FLAIR (hypointense on ADC (apparent diffusion coefficient) mapping) in the bilateral anterior circulation area (middle cerebral artery), resembling toxic edema (DWI, transverse; yellow arrows). **c** An atypically located intracerebral hemorrhage in the right frontal lobe on T2*-weighted MRI (transverse), also hyperintense on DWI (yellow ellipse in **b**, **c**)

**Fig. 4:**
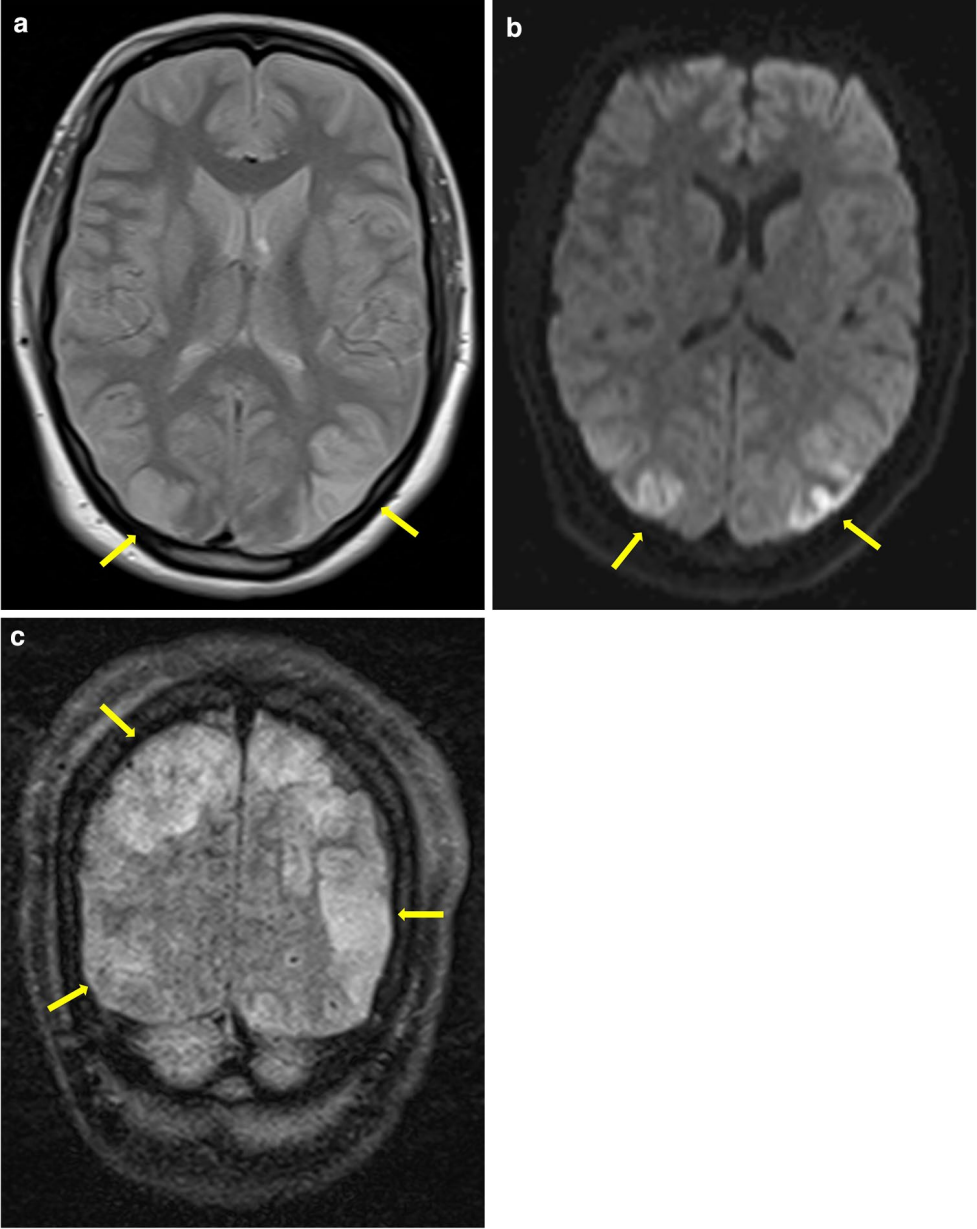
This figure represents the characteristic MRI findings of posterior eversible encephalopathy syndrome (PRES), one of the most common complications of reversible cerebral vasoconstriction syndrome (RCVS). **a**–**c** Bi-occipital hyperintensities in different T2-weighted images without ADC (apparent diffusion coefficient) hypointensities, corresponding to PRES (yellow arrows). **a** Proton-density-weighted (PDw) MRI in the transverse plane, **b** DWI in the transverse plane, and **c** FLAIR in the coronal plane

Overall, 30–55% of RCVS patients show no abnormalities on initial brain MRI, and 22% show no changes on MR angiography [4, 6, 40, 98]. This reflects the dynamics of vasoconstriction. In one-fourth of patients who have normal brain MRI on presentation, no MRI abnormalities emerge during the further course of the disease [98]. In the days to weeks following the initial TCH event, up to 81% of RCVS patients develop brain lesions: infarcts (39%), intracranial hemorrhage (ICH; 44%, including cSAH in 34% of cases and lobar hemorrhage in 20%), PRES (38%), or a combination thereof (Fig. 3) [4, 6, 86, 88]. Usually, risk factors do not predict lesion type, with the exception of pre-eclampsia, which is often associated with RCVS and PRES, two conditions that seem to share a common pathophysiology involving blood‒brain barrier breakdown and endothelial dysfunction [33, 91, 99]. This leads to passive extravasation of fluid and proteins, causing vasogenic edema [12, 33, 91]. PRES represents a clinical and radiological syndrome; in nearly 65% of cases, it features bilateral parieto-occipital accentuation (Figs. 3a, 4), involves the cortex and underlying white matter and is often reversible in a few days [100]. In addition, other structures, such as the basal ganglia, frontal and temporal cortex, and brainstem, may be affected by vasogenic edema [30]. PRES appears on FLAIR and T2-weighted imaging (Figs. 3a, 4) and in the form of hyperintensities on apparent diffusion coefficient (ADC) mapping [85]. PRES-like vasogenic edema is detected in 9–38% of RCVS patients, but approximately 85% of PRES patients also develop multifocal vasoconstriction of the small peripheral arterioles on conventional angiography due to impaired cerebral autoregulation and endothelial dysfunction with a primary perfusion deficit [4, 33, 101]. Thus, PRES may additionally lead to cytotoxic edema due to ischemic stroke or may even lead to hemorrhage [12]. It is even postulated that RCVS and PRES represent two syndromes of a common disease with impaired cerebral vascular tone and endothelial dysfunction, as they share strikingly common pathophysiology and clinical symptoms [33].

There is a marked temporal discrepancy between the occurrence of ischemic and hemorrhagic events [6, 40]. The severity of vasoconstriction peaks 9–13 days after the initial TCH event [6, 72]. Seventy-four percent of RCVS patients were found to have unusual severe headache approximately 9 days before ischemic stroke [72]. The process of vasoconstriction, caused by defective autoregulation of the vessel wall, begins in the distal capillary bed and migrates proximally [99]. This also reflects the dynamics of angiographic findings [102]. Thus, after approximately 2 weeks, the first and second segments of the great arteries become affected [99]. Maximal vasoconstriction (≥ 75%) in the P2 or M1 segment on MR-TOF angiography correlate significantly with PRES and ischemic stroke, respectively [86]. A study detected a mean of 4.52 stenoses per patient, which were fully reversible in 3–6 months [72]. In the clinical–radiological condition of RCVS, two infarct types are seen: smaller, peripheral, cortical infarcts, which occur rather early in the time course and are due to a primary perfusion deficit caused by maladaptive autoregulation in the arteriolar area, and larger, wedge-shaped watershed infarcts that are due to severe hypoperfusion distal to severe vasoconstriction (Fig. 3b) [11, 40, 98]. Ischemic strokes occur singly rather than in groups despite widespread vasoconstriction [98]. This further reflects the dynamics of the disease and prevents more pronounced infarction [98].

In general, the rate of hemorrhage as a complication is higher in RCVS than in PRES. Intracerebral hemorrhages occur in up to 44% of RCVS cases, with the most common form (22–35%) being convexity subarachnoid hemorrhage (cSAH) [4, 6, 40, 88]. Consequently, the most common complication of RCVS leading to hospitalization is ICH (Fig. 3c) [88]. Female gender and a history of migraine are considered independent risk factors for the occurrence of any type of hemorrhage in RCVS [40, 88]. Vascular aberrations start days before the attack and are characterized by dynamics of vasoconstriction and vasodilation [40, 99]. Abrupt stretching of the small pial cortical vessels may lead to reperfusion injury, which results in rupture of the small vessels and thus secondary hemorrhage [40, 88]. Hemorrhagic manifestations usually begin to appear 2–4 days after the initial TCH attack [40]. SAH in RCVS is usually confined to 1–3 sulci, overlying them, and is therefore overlooked in many cases [103, 104]. The most commonly affected site is the subarachnoid space over the frontal lobe (51%), followed by the anterior parietal region (21%) [104]. FLAIR-MRI characteristically shows what is known as the dot sign, which consists of punctate hyperintensities [98]. These hyperintensities are located in the area of the sulci of both hemispheres in RCVS patients and reflect the vasodilatation of the small cortical vessels [4, 98]. This finding supports the hypothesis that hemorrhages in RCVS are driven by cerebral autoregulation failure and sudden hypertension with resulting vasoconstriction and vasodilation, starting distally [90, 98].

### Differential diagnoses and neuroimaging findings

TCH is the cardinal symptom of RCVS and the main symptom of a ruptured brain aneurysm causing SAH [103]. As mentioned under the heading "Complications", RCVS itself can lead to subarachnoid hemorrhage [40]. However, in up to 85% of patients, SAH is a consequence of aneurysm rupture [6, 105]. A less common cause of SAH may be RCVS itself. In addition, it must be mentioned that unruptured aneurysms may occur in 3–8% by chance [103, 106]. An important and difficult point emerges, namely, the distinction between SAH secondary to RCVS and SAH due to a ruptured aneurysm (aSAH) [107]. This distinction is of enormous relevance, because RCVS is actually a benign entity, in contrast to aneurysm, a more life-threatening cause of SAH. A major distinguishing feature of both entities is that the blood component in the subarachnoid space due to RCVS is unilaterally localized, covering only 1–3 adjacent sulci in up to 83% of patients in a retrospective study by Kumar et al. [104]. When SAH is localized in this way, it is sometimes referred to as cortical or convexity SAH (cSAH) [6, 105, 107, 108]. In the context of aSAH, blood is distributed across a wider range of locations, including the sylvian fissure and basal cisterns, and ventricular collapse can also be observed [103]. Vasospasm due to aSAH usually occurs unilaterally around the SAH and involves 1–2 medium-sized arteries [107]. Long, smooth regions of vasoconstriction, primarily of the proximal cisternal segments, usually peak from day 4 to day 14 after aneurysm rupture, in contrast to vasospasm in RCVS, which is widely distributed before cSAH occurs [107]. In addition, it seems unlikely that the amount of subarachnoid blood in aSAH can cause vasospasm scattered throughout the brain vessels. This is in contrast to RCVS, in which there is often minimal subarachnoid blood content, but vasospasm is widespread and multifocal with involvement of bilateral arteries [109]. Furthermore, approximately 80% of aSAH patients develop only one episode of TCH, whereas 85–100% of RCVS patients develop multiple episodes (up to 4) in the first few days [103].

**Fig. 5:**
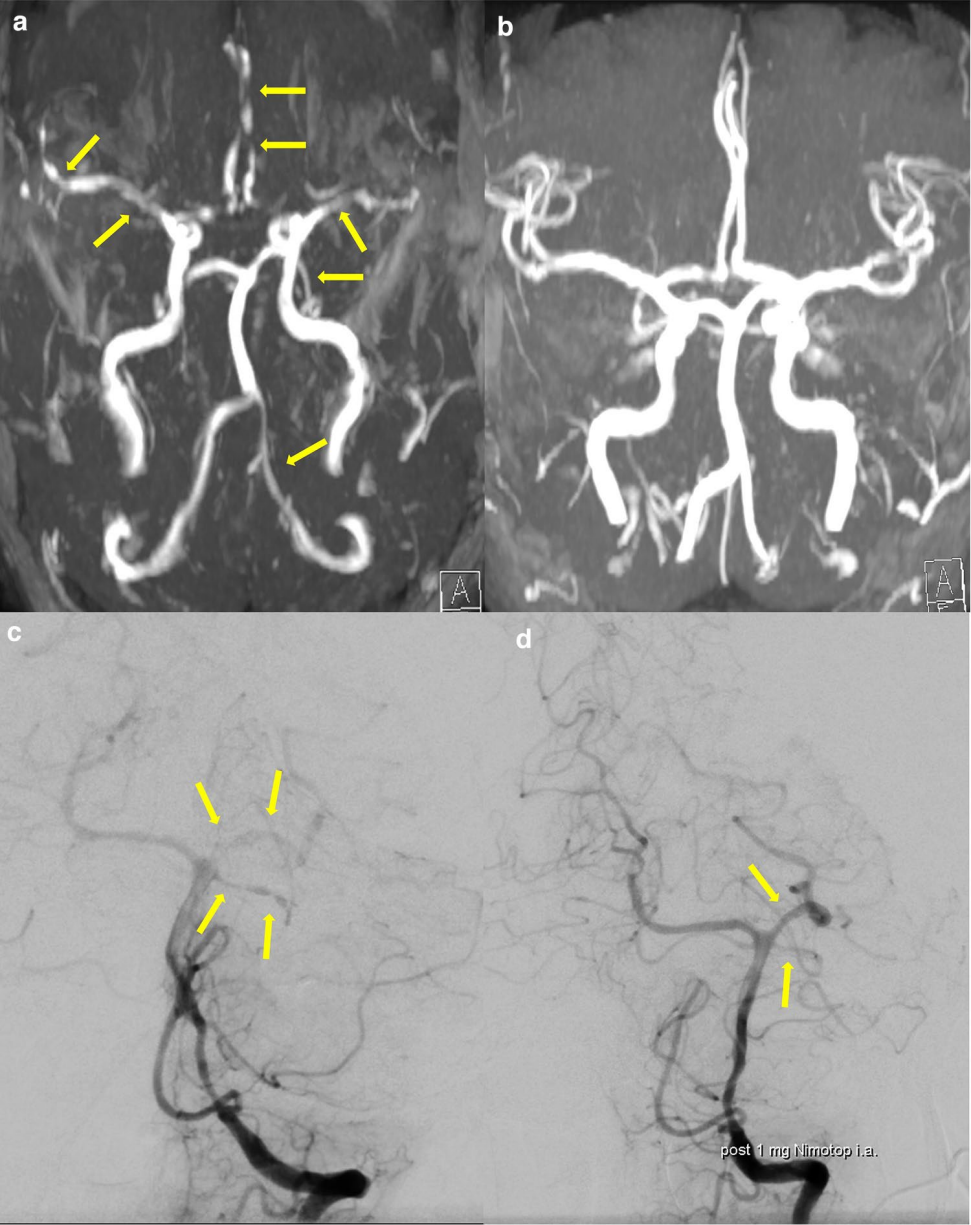
Typical angiographic findings in the patient with reversible cerebral vasoconstriction syndrome (RCVS) shown in Fig. 3. **a** MR-TOF angiography with diffuse regions of vasoconstriction (creating a “sausage-on-a-string” appearance) in all territories (yellow arrows). **b** Full remission of vasoconstrictions on MR-TOF angiography 3 weeks after an oral nimodipine regimen was started. **c** Digital subtraction angiography (DSA) via left vertebral artery in the same patient. Multiple vasospasms in the area of the left posterior cerebral artery and left superior cerebellar artery (yellow arrows) as well as in the left anterior cerebral artery (not shown) can be detected. **d** Diagnostic DSA after intra-arterial administration of 1 mg nimodipine. Complete reversibility of the vasospasms was observed

On the basis of vascular stenoses, one can identify another differential diagnosis, namely, primary angiitis of the central nervous system (PACNS) [110]. In contrast to the severe, smooth, tapering, widespread vasoconstrictions, and vasodilatations of the vessels of the circle of Willis primarily after passage through the dura in RCVS (Fig. 5a), the irregular, notched, eccentric stenoses and ectasias in PACNS result from chronic vessel wall inflammation and destruction with distal cut-offs [11, 98, 111, 112]. Vasoconstriction in RCVS is reversible, which is also reflected in the established diagnostic criteria (Table 2) [11]. Whereas digital subtraction angiography (DSA) often fails to show any abnormalities in PACNS, it is always abnormal in RCVS (Fig. 5c) [11, 98, 111]. The reversibility of stenoses is an important point of differentiation from PACNS, which is why calcium channel antagonists can also be given diagnostically in the context of conventional angiography (Fig. 5d) [9, 10, 113]. PACNS is accompanied by dull persistent headache in 51% of cases [98]; TCH occurs in only approximately 6% [98]. The analysis of cerebrospinal fluid (CSF) is an indispensable first diagnostic tool for headache diagnosis [114]. Patients with PACNS present with pathological CSF findings in up to 90% of cases [4, 111]. Important alterations include moderate pleocytosis with an increased protein concentration, occasional intrathecal IgG synthesis, and the appearance of oligoclonal bands [111]. However, abnormal CSF findings are atypical in RCVS and, if present, include minimal pleocytosis and slightly elevated protein levels [110, 114]. In a retrospective study by Singhal et al. comparing PACNS and RCVS patients in different aspects, such as symptoms and brain and angiographic findings, one-quarter of all RCVS patients showed no abnormalities regarding the brain parenchyma on initial MRI despite widespread and severe vessel narrowing [98]. Moreover, RCVS patients were significantly younger, had a 2.6-fold higher proportion of women, and had a higher rate of recent intake of vasoconstrictive or illicit drugs [98]. Finally, RCVS patients are more likely to develop wedge-shaped infarcts in watershed regions, in contrast to PACNS patients, nearly 76% of whom have small and widespread acute, subacute, or chronic infarcts with diffuse T2-hyperintense white matter lesions [98, 111, 112]. Only approximately 2–9% of PACNS patients develop parenchymal hemorrhage or cSAH, whereas PRES has not yet been reported in PACNS [98]. The brainstem and deep gray matter are involved in up to 74% of infarcts in PACNS, which rarely have a predilection for ischemia in RCVS [98]. The most important distinguishing diagnostic features are summarized in Table 5.

**Table 5:** Different causes for vessel narrowing in neurological disorders and their main diagnostic findings [4, 11, 98, 103, 104, 107, 109–112, 114]

Variable	RCVS	PACNS	SAH-induced vasospasm
CSF findings	Usually normal or mild pleocytosis and slightly elevated protein levels	Abnormal in 80–90% with moderate pleocytosis, elevated protein levels, OCB, intrathecal IgG synthesis	Abnormal: elevated red blood cell count, xanthochromia
Brain imaging	Possible findings: none; ischemic stroke (mainly border zone); hemorrhages (ICB, cSAH: focal, overlying the cortical convexity, often only 1–3 sulci involved); parieto-occipital stressed vasogenic edema (PRES)	Widely distributed acute/subacute/chronic infarcts in gray/white matter and brainstem, possible diffuse white matter lesions	SAH: blood in the sylvian fissure and the basal cisterns
Vascular imaging	Widespread, symmetrical, severe, tapering vasoconstriction and vasodilatation concerning the circle of Willis (“string-and-beads”); reversible	Normal; irregular, eccentric, notched with distal cut-offs; irreversible	Possible (ruptured) aneurysm or arteriovenous-malformation, smooth, long, segmental narrowing (esp. proximal cisternal segments), occurring after 4–14 days, expansion in correlation with SAH; reversible

*RCVS* reversible cerebral vasoconstriction syndrome, *PACNS* primary angiitis of the central nervous system, *SAH* subarachnoid hemorrhage, *OCB* oligoclonal bands, *CSF* cerebrospinal fluid findings, *ICB* intracerebral hemorrhage, *cSAH* convexity subarachnoid hemorrhage, *PRES* posterior reversible encephalopathy syndrome

The modified Rankin scale (mRS) score at discharge was between 0 and 1 in 90% of RCVS patients, which was significantly higher than the proportion in PACNS patients (70%) [98]. To date, there are no official guidelines for RCVS therapy. Many case reports and small prospective studies showed that the use of calcium channel antagonists (nimodipine, nifedipine, or verapamil) as early as possible could have favorable effects on the clinical course (Fig. 5b) [1, 4, 8, 9, 18, 115, 116]. In a prospective study by Cho et al., 86.6% of clinically and radiologically confirmed RCVS patients achieved remission of TCH [7].

Patients are often subjected to risky diagnostics, especially biopsies of the vessel wall, or are started on immunosuppressive therapy, primarily with glucocorticoids, out of concern that PACNS may be overlooked [98, 110]. In a study, PACNS and RCVS patients were compared in terms of clinical, laboratory, and radiological aspects [98]. Criteria for rapid bedside diagnosis were developed. The authors reported that these criteria achieved a specificity and a positive predictive value of 100% for RCVS in patients with recurrent TCH episodes or a single TCH episode combined with either border zone infarcts, evidence of PRES, or even normal intracranial imaging [98]. In contrast, inflammatory markers in CSF plus infarcts in the brainstem or deep gray matter achieve a positive predictive value of 100% for PACNS [98]. A detailed differential diagnosis is also important with regard to the upcoming therapeutic options. It has already been demonstrated that treatment with glucocorticoids in RCVS patients is an independent predictor of clinical–radiological deterioration with a poor outcome [4, 37, 117]. Clinical worsening was associated with new infarcts in 44–70% of patients. Independently, the occurrence of ischemic infarcts in baseline imaging is a predictor of poor outcome [38]. Forty-seven percent of glucocorticoid-treated RCVS patients had an mRS of 4–6 at discharge, compared to 17% in the untreated group [37]. One case report even discussed low-dose steroid treatment in the context of rheumatoid arthritis as a precipitating factor for RCVS [18].

## Prognosis and conclusion

Despite the occurrence of serious complications, the prognosis of RCVS in both adults and children is very good. Over 90% of patients have a good outcome, defined as 0–1 on the mRS [4, 43, 98]. A worse outcome is associated with severe infarcts early in the disease history and pronounced focal neurological deficits at baseline, leading to death [38]. As mentioned above, glucocorticoids worsen the outcome; in contrast, vasoactive agents such as SSRIs and triptans are associated with worsening vasospasm or new neurological deficits, underlining the role of serotonergic drugs in the pathophysiology, while these agents do not worsen the clinical outcome [4, 37]. Overall, 97.5% of patients in a retrospective study were functionally independent according to the Barthel index [118]. Even after TCH was no longer present and vasoconstriction was reversed, 53% of patients continued to have mild-to-moderate headache. Of these, the majority were triggered by migraine and vasoactive medication [118].

However, the pathophysiology is still not fully understood. Further basic studies are necessary for a better understanding and possibly for further therapeutic options, as some fulminant cases, e.g., in the postpartum state, can still lead to death or severe disabilities [47].

## Supplementary Information

Below is the link to the electronic supplementary material.

Supplementary file1 (PDF 84 KB)

## Data Availability

The material used during the current work is available from the corresponding author on reasonable request.

## Abbreviations

TCH: Thunderclap headache
RCVS: Reversible cerebral vasoconstriction syndrome
PRES: Posterior reversible encephalopathy syndrome
CT: Computer tomography
MRI: Magnetic resonance tomography
PACNS: Primary angiitis of the central nervous system
SAH: Subarachnoid hemorrhage
cSAH: Convexity subarachnoid hemorrhage
aSAH: Subarachnoid hemorrhage due to aneurysm rupture
ICH: Intracranial hemorrhage
eNOS: Endothelial nitrite oxide synthase
PGI2: Prostacyclin
BBB: Blood–brain barrier
SSRI: Selective serotonin reuptake inhibitors
SNRI: Serotonin and noradrenaline reuptake inhibitors
PPA: Postpartum angiopathy
ACE2: Angiotensin-converting-enzyme 2
(CE-) FLAIR: (Contrast-enhanced) fluid-attenuated inversion recovery
BDNF: Brain-derived neurotrophic factor
ICDH-3: International Classification of Headache Disorders 3 (beta version) criteria
CSF: Cerebrospinal fluid
OCB: Oligoclonal bands
mRS: Modified Rankin scale
