# Supplementary material for: RCVS: by clinicians for clinicians—a narrative review
Source: J Neurol. 2022 Oct 28;270(2):673–88. doi: 10.1007/s00415-022-11425-z (PMC9615630; doi:10.1007/s00415-022-11425-z)
Supplement: Supplementary file 1 — Supplementary file1 (PDF 84 KB) [file 415_2022_11425_MOESM1_ESM.pdf]

RCVS – by clinicians for clinicians: a narrative review

Journal of Neurology

Deborah Katharina Erhart[1]*, Albert Christian Ludolph[1], Katharina Althaus[1]

[1]University of Ulm, Department of Neurology, Oberer Eselsberg 45, 89081 Ulm, Germany
*Corresponding author: Deborah.Erhart@rku.de

Author/year/reference	Cases/sex (m/f)	Risk factor/trigger	Clinical features	Complications
Liao et al., 2003 [55]	4 (-/4)	Bathing	TCH (all)	Small cerebellar ischemia and PRES (one patient)
Mak et al., 2005 [62]	6 (-/6)	Bathing	TCH (all)	Small ischemia in both frontal lobes (one patient)
Kato et al., 2018 [63]	1 (-/1)	Coughing	TCH	Cerebral infarcts in both parietal lobes
Theeler et al., 2010 [20]	1 (1/-)	Sexual activity	TCH	None
Komatsu et al., 2014 [19]	1 (-/1)	Physical exertion, coughing, Valsalva maneuver	TCH, transient homonymous left hemianopsia, facial sensory deficit	cSAH
Wong et al., 2009 [64]	2 (-/2)	1.+2. Physical exertion, THC	1. Recurrent TCH<br>2. TCH, transient dysphasia and left leg paresis	1. Atypically located ICH, SDH<br>2. None

Uhegwu et al., 2015 [21]	1 (1/-)	THC	Right hemiparesis, aphasia, left gaze deviation	Cerebral infarcts located in the left thalamus and left parietal lobe
Roberts et Sowers, 2020 [22]	1 (1/-)	THC	Recurrent TCH	None
Hu et al., 2010 [65]	1 (-/1)	Amphetamine derivatives, postpartum, sexual activity	TCH	None
Marder et al., 2012 [23]	6 (1/5)	1. Traumatic brain injury<br>2. Amphetamine derivatives, THC<br>3. Caffeine, nicotine<br>4. Bathing, triptans, ergotamine<br>5. SSRI<br>6. SSRI	1. Left-sided hemiplegia<br>2. Disorientation, ataxia<br>3. Disorientation<br>4. Recurrent TCH<br>5. TCH<br>6. TCH	1. cerebral infarction in both anterior circulations, atypically located ICH<br>2. cSAH<br>3. Atypically located ICH, SDH<br>4. Atypically located ICH, SDH<br>5. cSAH<br>6. SAH, atypically located
Baharith et Zarrin, 2016 [24]	1 (-/1)	Khat	Right-sided hemiparesis, aphasia	Ischemic infarct in the left frontal lobe
Singhal et al., 2002 [13]	3 (1/2)	1. SSRI<br>2. SSRI<br>3. Triptan	1. Right-hemianopsia<br>2. Weakness of the left hand<br>3. Generalized seizure	1. Cerebral infarction in the parieto-occipital lobe<br>2. watershed infarcts<br>3. watershed infarcts

John et al., 2013 [49]	2 (-/2)	1. SSRI, THC, ergotamine, hydromorphone<br>2. Combination of SDNRI and SSRI	1. TCH, disturbance of consciousness up to coma<br>2. Serotonergic syndrome, TCH, generalized seizure, left-sided hemiparesis	1. PRES, SAH, cerebral infarcts in the right cerebellum and in both frontal-parietal lobes<br>2. Cerebral infarcts in both parietal lobes
Manning et al., 2021 [51]	1 (-/1)	SSRI	Left-sided hemiparesis	Multiple right-stressed cerebral infarcts
Zeitouni et al., 2021 [69]	1 (-/1)	SNRI	TCH, left-sided hemiparesis	Cerebral infarcts in the right anterior and posterior circulation
Koopman et al., 2008 [73]	2 (-/2)	1. THC<br>2. THC, triptans	1. Left-sided hemiparesis<br>2. TCH	1. Multiple right-stressed cerebral infarcts<br>2. None
Meschia et al., 1998 [14]	1 (1/-)	Triptans, corticosteroids	TCH, right homonymous hemianopsia	Cerebral infarct in the left occipital lobe
Kato et al., 2016 [50]	2 (-/2)	1. Triptans<br>2. Triptans, postpartum	1. TCH<br>2. TCH	1. SAH, PRES<br>2. None
Palma et al., 2012 [26]	1 (-/1)	Epinephrine	Recurrent TCH, blurred vision	Cerebral infarct in the right occipital lobe
Calic et al., 2014 [25]	1 (-1)	Indomethacin	Recurrent TCH	None
Kraemer et al., 2015 [54]	1 (-/1)	Fingolimod, high-dose corticosteroids	Recurrent TCH, left hemianopsia	Cerebral infarct in the right occipital lobe
Erhart et al., 2022 [18]	1 (-/1)	Low-dose corticosteroids	Aphasia, left leg-stressed hemiparesis	Multiple cerebral infarctions in the anterior circulation
Moustafa et al, 2009 [109]	3 (-/3)	1. Phytoestrogens	1. TCH	1. SAH<br>2. cSAH

		2. Nasal decongestants (pseudoephedrine)	2. TCH, paresis of the right hand<br>3. TCH	3. None
Cantu et al., 2003 [70]	22 (10/12)	Nasal decongestants (pseudoephedrine, phenylpropanolamine)	TCH +/- focal neurological deficits	Ischemic stroke (1/21), ICH (16/22), SAH (4/22), ICH + SAH (1/22)
Doss-Esper et al., 2006 [57]	1 (-/1)	Intravenous immunoglobulins	TCH, cortical blindness, confusion	PRES, cSAH, multiple bilateral ischemic strokes
Saito et al, 2019 [59]	1 (-/1)	Blood transfusion	Coma (hemorrhagic shock)	PRES
Boughammoura et al., 2003 [58]	1 (-/1)	Blood transfusion	Repeated focal and generalized seizures, status epilepticus	cSAH
Ray et al., 2022 [52]	1 (-/1)	COVID-19	Quadriparesis, focal seizures, blurred vision	PRES
Mansoor et al., 2021 [81]	1 (-/1)	COVID-19	TCH, blurred vision	PRES
Dakay et al., 2020 [82]	1 (-/1)	COVID-19, vertebral artery dissection	TCH	cSAH
Singhal et al., 2004 [12]	4 (-/1)	1. Postpartum<br>2. Postpartum, SSRI<br>3. THC<br>4. Postpartum	1. TCH<br>2. TCH, generalized seizures<br>3. TCH, generalized seizures<br>4. TCH	1. PRES<br>2. PRES, cSAH<br>3. PRES<br>4. PRES
Feil et al., 2017 [33]	1 (-/1)	Postpartum, CSF leakage	Serial generalized seizures, coma	PRES
Fugate et al., 2012 [47]	4 (-/4)	1. Postpartum<br>2. Postpartum	1. TCH, paresis oft left arm and right leg	1. cSAH, ICH, multiple cerebral infarcts

		3. Postpartum eclampsia<br>4. Postpartum eclampsia	2. TCH, left hemiplegia, left hemineglect<br>3. Drowsiness, confusion<br>4. TCH, Generalized seizure	2. ICH in the right basal ganglia<br>3. Global edema<br>4. Global edema
Hadhiah et al., 2021 [48]	1 (-/1)	Postpartum	TCH, generalized seizures	cSAH
Singhal et al., 2009 [74]	1 (-/1)	Postpartum eclampsia	TCH, right hemiparesis	PRES, ischemic stroke in the left frontal lobe, ICH in the right frontal lobe
Pop et al., 2019 [35]	1 (-/1)	Postpartum eclampsia	TCH, seizures	PRES
English et Nasr, 2019 [68]	1 (-/1)	Pheochromocytoma	Recurrent TCH	None
Togha et al., 2021 [60]	1 (-/1)	Pheochromocytoma	Recurrent TCH	Small ICH in the right parietal lobe
Verillaud et al., 2010 [61]	2 (1/1)	Carotid glomus tumor +/- nasal decongestants	Recurrent TCH	None
Lopez-Valdes et al., 1997 [27]	2 (1/1)	Carotid surgery (both)	1. TCH, left hemianopsia, transient left hemiparesis<br>2. TCH, left hemianopsia, left hemineglect	1. Cerebral infarcts in the right frontal and occipital lobes<br>2. Ischemic stroke in the right parietal and occipital lobes
Rosenbloom et Singhal, 2007 [28]	1 (-/1)	Carotid surgery, Valsalva maneuver	TCH, transient aphasia and paresis of the right hand	Multiple cerebral infarcts
Noda et al., 2011 [106]	1 (-/1)	Unruptured aneurysm	TCH, generalized seizures, paresis of the left lower limb	cSAH, PRES, ischemic stroke in the right frontal lobe and in both parietal lobes

Online Resource 1 Case reports and case series describing risk factors and triggers of reversible cerebral vasoconstriction syndrome (RCVS), including their specialties. The numbers of the references refer to the bibliography in the main text.

Abbr.: TCH: thunderclap headache, PRES: posterior reversible vasoconstriction syndrome, SAH: subarachnoid hemorrhage, cSAH: convexity subarachnoid hemorrhage, SDH: subdural hemorrhage, ICH: intracranial hemorrhage, THC: tetrahydrocannabinol, SSRI: selective serotonin reuptake inhibitors, SNRI: serotonin and noradrenaline reuptake inhibitors, SDNRI: serotonin, dopamine and noradrenaline reuptake inhibitors, CSF: cerebrospinal fluid
